# Cranial stent position is independently associated with the development of TIPS dysfunction

**DOI:** 10.1038/s41598-022-07595-5

**Published:** 2022-03-03

**Authors:** Carsten Meyer, Alba Maria Paar Pérez, Johannes Chang, Alois Martin Sprinkart, Nina Böhling, Andreas Minh Luu, Daniel Kütting, Christian Jansen, Julian Luetkens, Leon Marcel Bischoff, Ulrike Attenberger, Christian P. Strassburg, Jonel Trebicka, Karsten Wolter, Michael Praktiknjo

**Affiliations:** 1 Department of Radiology, University Hospital Bonn, Bonn, Germany; 2 Department of Internal Medicine I, University Hospital Bonn, Venusberg-Campus 1, 53127 Bonn, Germany; 3 Department of General and Visceral Surgery, St. Josef Hospital, University of Bochum, Bochum, Germany; 4 Department of Internal Medicine 1, University of Frankfurt, Frankfurt, Germany; 5 European Foundation for the Study of Chronic Liver Failure - EF CLIF, Barcelona, Spain

**Keywords:** Liver cirrhosis, Portal hypertension

## Abstract

Complications of portal hypertension can be treated with transjugular intrahepatic portosystemic shunt (TIPS) in selected patients. TIPS dysfunction is a relevant clinical problem. This study investigated the prognostic value of two-dimensional (2D) TIPS geometry for the development of TIPS dysfunction. Three hundred and seven patients undergoing TIPS procedure between 2014 and 2019 were analyzed in this monocentric retrospective study. 2D angiograms from the patients with and without TIPS dysfunction were reviewed to determine geometric characteristics including insertion and curve angles and the location of the stent. Primary outcome was the development of TIPS dysfunction. A total of 70 patients developed TIPS dysfunction and were compared to the dysfunction-free (n = 237) patients. The position of the cranial stent end in the hepatic vein and the persistence of spontaneous portosystemic shunts were significantly associated with the development of TIPS dysfunction. Among significant parameters in univariable regression analysis (portal vein-pressure after TIPS, Child–Pugh Score before TIPS, MELD before TIPS and white blood cell count before TIPS), multivariable models showed cranial stent position (p = 0.027, HR 2.300, 95% CI 1.101–4.806) and SPSS embolization (p = 0.006, HR 0.319, 95% CI 0.140–0.725) as the only predictors of TIPS dysfunction. This monocentric study demonstrates that the position of the cranial stent end is independently associated with the development of TIPS dysfunction. The distance of the cranial stent end to the IVC at the time of TIPS placement should be less than 1 cm in 2D angiography.

## Introduction

Liver cirrhosis is a major health care burden. A variety of severe complications of portal hypertension such as variceal bleeding and refractory ascites, lead to high hospitalization rates and increased morbidity and mortality^1^.

These severe complications of portal hypertension can be treated by implantation of a transjugular intrahepatic portosystemic shunt (TIPS), which partially redirects the portal venous blood flow to the inferior vena cava (IVC) and thereby reduces the portosystemic pressure gradient^2^. In selected patients, TIPS can improve outcome of patients with decompensated cirrhosis^3–7^.

One of the main complications was TIPS dysfunction in up to 80% of all patients within 2 years, in the old era of bare metal stents^8,9^. After years of resolving mostly technical problems, the introduction of polytetrafluoroethylene (PTFE) covered stents in the millennials marked an important development with a reduction but not abolishment of shunt dysfunction, even in smaller stent diameters^2,10–14^. TIPS dysfunction, stenosis and occlusion, among other factors^15–18^, seem to be influenced by the hemodynamic flow characteristics^19^, which themselves are influenced by the geometry of the TIPS stent. In the modern era of PTFE-covered TIPS stents, some studies suggested that characteristics of TIPS stent geometry, such as portal venous inflow, retrieved by two-dimensional (2D) angiography during the procedure might predict TIPS dysfunction^20,21^. Some smaller series suggested an association of the landing zone of TIPS stent in the portal or hepatic vein with the development of TIPS dysfunction. The placement of the TIPS stent in the hepatic vein to IVC junction seems important to reduce the risk of hepatic venous stenosis or occlusion^17,22–28^. Moreover, interventional embolization of spontaneous portosystemic shunts (SPSS) during TIPS procedure was associated with less episodes of hepatic encephalopathy^29^. Larger cohort data on predicting the development of TIPS dysfunction as early as at the time of TIPS placement is still scarce, given the potentially devastating effects of recurrent variceal bleeding and ascites.

Therefore, the aim of this study was to determine whether the 2D TIPS geometrical characteristics at the time of TIPS creation can predict TIPS dysfunction in a large cohort.

## Methods

### Study population

**Figure 1:**
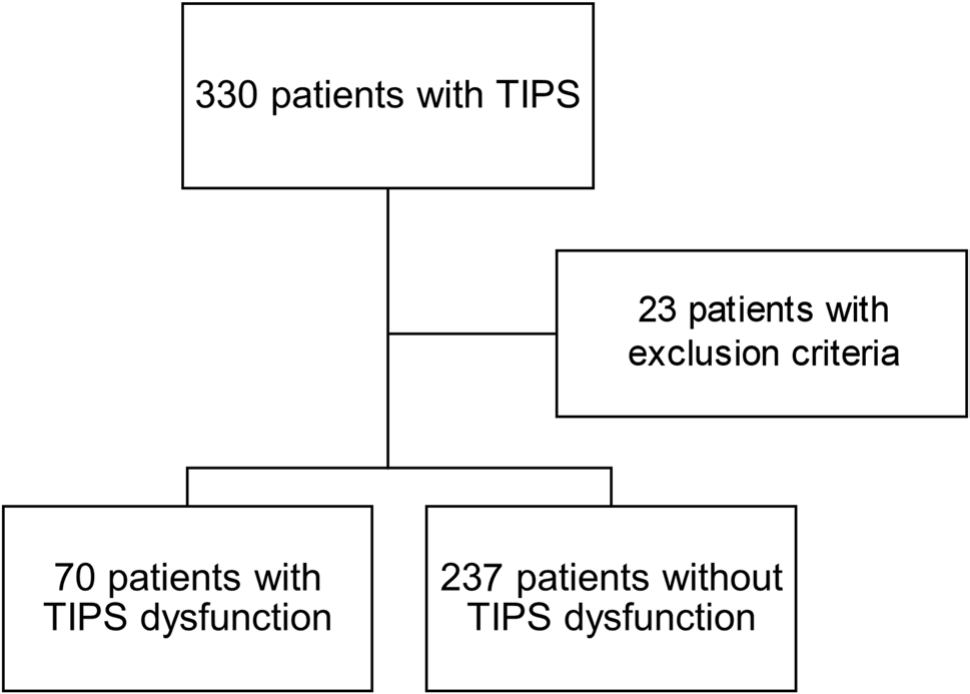
Flow chart of study population: Study population at baseline with 330 patients, exclusion of 27 patients due to interventional TIPS reduction, final 303 patients, thereof 70 with TIPS dysfunction and 233 without dysfunction.

This is a retrospective analysis of our observational monocenter NEPTUN and NEPTUN 2 cohorts (Non-invasive Evaluation Program for TIPS and Follow Up Network) (clinicaltrials.gov identifier: NCT03628807 and NCT04393519) of patients undergoing TIPS procedure between January 1, 2014, to December 31, 2019 in our institution. The patients were regularly followed up clinically every 3 to 6 months using non-invasive imaging such as ultrasound and computer tomography (CT) as well as standard laboratory biochemical blood analyses to evaluate TIPS function (Fig. 1).

Inclusion criteria were patient age older than 18 years, first-time treatment with a TIPS, implantation of a PTFE-covered TIPS stent (Viatorr) and available digital subtraction angiography (DSA) studies from the time of TIPS placement. Exclusion criteria were previous TIPS revisions including balloon dilatation, stent-in-stent placement, and local lysis of the TIPS tract as well as reduction of TIPS stent. Patients with non-cirrhotic splanchnic venous thrombosis due to prothrombotic vascular liver disease have a different clinical trajectory. Thus, they were excluded from the study. All patients received anticoagulation with heparin or low molecular weight heparin (Partial Thromboplastin Time 2–3 times above normal) for 7 days without subsequent anticoagulation according to our institutions standard protocol.

Primary outcome was first revision due to TIPS dysfunction, defined as abnormal duplex sonographic measurement (reduction of flow velocity of more than 50% or missing flow), ascites, bleeding, or progression of esophageal varices, with resulting invasive revision of TIPS.

All patients signed written informed consent. This study was performed according to the guidelines of the Helsinki Declaration. Local ethics committee (Ethikkomitee der Medizinischen Fakultät, Universität Bonn) approved this study (Lfd. Nr. 038/20).

### TIPS procedure

The TIPS procedure was performed by a team of experienced radiologists and hepatologists under fluoroscopic and ultrasound guidance as previously described^14^. All contraindications were precluded beforehand. The procedure was performed under analgesia with pethidine. Initial portosystemic pressure gradient (PSPG) was recorded, then the 8–10 mm nominal diameter covered TIPS stent (Gore Viatorr endoprothesis, W.L. Gore Medical) was implanted. The TIPS stent was dilated according to PSPG at interventionalist’s discretion. Post-TIPS PSPG targets were PSPG < 12 mmHg for variceal bleeding or 50% PSPG reduction for refractory ascites. Length of the stent was calculated by 2D angiogram with measuring pig tail catheter. SPSS, if present, were embolized with coils or histoacryl according to the interventionalist’s discretion. TIPS patients were followed by routine follow ups including ultrasound examinations in our outpatient clinic every 3–6 months.

### Assessment of two-dimensional TIPS geometry

Commercially available clinical imaging systems (Philips Allura Clarity and Philips Intellispace; Philips Healthcare GmbH,) were used to analyze the geometrical data of the TIPS tract. A completion portogram (2D DSA projection in patient inspiration) was used for measurement of the stent location. All angles were measured with Impax EE (Agfa Healthcare GmbH,) in degrees, distances were measured in cm. The angles of the TIPS tract were determined on those 2D images (all anterior–posterior) retrieved. If SPSS appeared during contrast medium application, they were classified into umbilical, coronary, splenorenal, and other based on their course. According to the measured diameter at the visibly widest point in DSA projection, SPSS were subsequently classified into the largest, second largest and third largest shunt. If embolization of the shunt was performed, either by coils, histoacryl, or both, it was documented.

**Figure 2:**
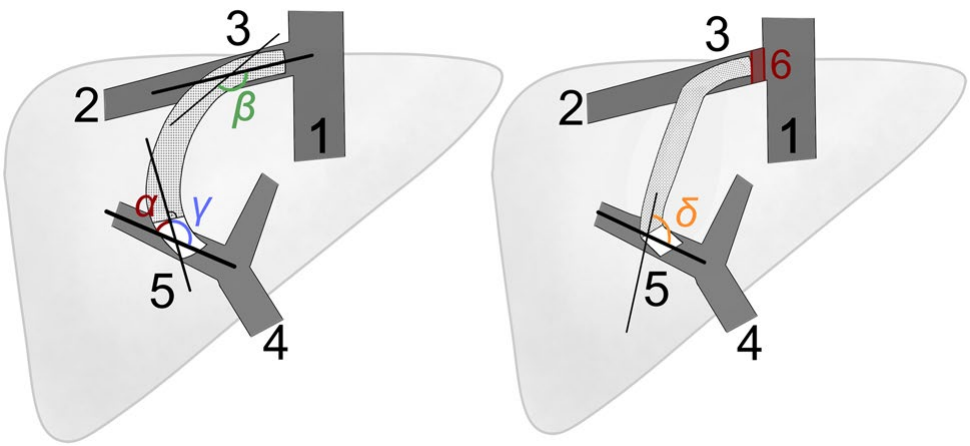
Schematic presentation of the measured two-dimensional TIPS stent geometry parameters and angles: 1: IVC; 2: liver vein; 3: cranial TIPS end; 4: PV, 5: distal TIPS end 6: distance between cranial TIPS stent end and IVC measured in cm (stent ends that extended into the IVC were noted as ≤ 0 cm); α- and γ-angle: angles measured between a straight line drawn at right angles to the beginning of the covered stent and a second straight line passing through the middle of the PV; β-angle: angle between the hepatic vein and the cranial TIPS stent end continued course; δ-angle: angle measured between the course of the PV and the course of the lower TIPS tract.

The following geometric characteristics have been defined as illustrated in Fig. 2, created with Krita (version 4.2.8, KDE).

#### Cranial TIPS stent end

The distance between cranial stent end and inferior vena cava (IVC) in cm was measured with the initially placed radio-opaquely marked pigtail catheter as reference. Distance of more than 1 cm from the IVC was defined as the cranial TIPS stent ending in the hepatic vein. ROC Analysis was performed for the distance of cranial stent end to IVC (AUC = 0.592), and Youden Index showed an optimal cut off between 0.9 cm and 1.1 cm. Thus, we chose 1 cm as cut off.

#### α and γ angle

Supplement angles between two straight lines: the first runs orthogonally to and centrally through the beginning of the covered stent part (defined by the radiopaque gold ring of the TIPS stent). The second straight line models the course of the portal vein and runs centrally through a section of the vessel chosen to be as long as possible, in the middle of which the TIPS stent debouches.

#### β angle

Angle between a straight line projecting the cranial course of the TIPS stent and oriented to radiopaque markings of the stent and a straight line extending to behind the cranial stent entry site and representing the course of the hepatic vein by a centrally located line.

#### δ angle

Angle between the projected course of the lower TIPS tract, represented by a straight line passing centrally through the stent section that lies immediately cranial to the inferior confluent vessel, and a straight line modeling the course of the portal vein as described in α and γ angle.

#### IVC reflux

Reflux of contrast media after TIPS stent implantation was analyzed in angiogram loops.

#### Retrograde intrahepatic portal venous flow

Angiogram loops were analyzed for retrograde flow in the intrahepatic portal venous branches after TIPS implantation.

#### Caudal TIPS stent end

Determined on the final images after TIPS implantation and classified as insertion in portal vein, exact intersection of portal vein and liver parenchyma or liver parenchyma.

All measurements were performed manually by both an expert radiologist and trained hepatologist.

### Statistical analysis

Descriptive statistics were run for all variables. Continuous variables are shown as median (range), categorial variables as percentage or absolute cases. To compare the dysfunction and the dysfunction-free groups, non-parametric testing was used. Uni- and multivariable regression models were used to identify predictors of TIPS dysfunction, p values below 0.05 were considered statistically significant. Interobserver agreement was determined by Intraclass Correlation Coefficient (ICC) and Cohen's Kappa. ICC was used for variables with quantitative measurement scales and Cohen's kappa for variables with categorical measurement scales; values above 0.8 were considered good and values above 0.9 were considered excellent interobserver agreement. Analysis of all was performed with Statistical Package for Social Sciences (SPSS version 24, IBM,).

## Results

### General patient characteristics at baseline

This study included three hundred and seven patients (n = 184 (60%) male) who underwent TIPS procedure. In 90% of our patients, TIPS was created between the right hepatic and right portal veins. Median age at TIPS procedure was fifty-nine (18–87) years. The most frequent indication for TIPS was refractory ascites in one hundred and ninety-one cases (63%); one hundred and twelve (37%) TIPS were implanted for variceal bleeding. The two main causes of cirrhosis were alcohol-related (n = 184, 60%) and chronic viral hepatitis (n = 37, 12%).

Seventy (23%) patients developed TIPS dysfunction (Fig. 1). Median time to TIPS dysfunction was six (0–84) months. The most frequent indications for revision were signs of dysfunction on duplex-sonography and/or clinical reoccurrence of ascites (n = 54, 82%). There were no significant differences in procedure-related complication rates (Supplementary Table 1).

For local factors such as hepatocellular carcinoma (HCC) and for hepatic hemodynamics before and after TIPS procedure, there was no significant difference between the dysfunction and dysfunction-free group (Table 1). Model of end-stage liver disease (MELD) score before TIPS (12 (6–27) dysfunction-free group vs 11 (6–21) dysfunction group; p = 0.036) and Child–Pugh score before TIPS (9 (5–14) dysfunction-free group vs 9 (5–13) dysfunction group; p = 0.041) showed a significant difference between the two groups.

**Table 1:** General Characteristics at baseline & outcome.

Parameter		No dysfunction (n = 237)	TIPS dysfunction (n = 70)	p
General	Age (years)	59 (18–87)	59 (23–78)	0.638
	Sex (male/female)	145/92 (61%/39%)	39/31 (56%/44%)	0.413
	Indication for TIPS^a^ (variceal bleeding/refractory ascites)	89/144 (38%/62%)	23/47 (33%/67%)	0.336
	Ethiology of cirrhosis (alcohol/viral/other)	148/25/64 (62%/11%/27%)	36/12/22 (51.5%/17%/31.5%)	0.236
	State follow up (alive/dead/liver transplantation) total	154/78/5 (65%/33%/2%)	45/24/1 (64%/34%/2%)	0.946
	HCC^b^ before TIPS (yes)	8 (3%)	1 (1%)	0.387
	HCC developed after TIPS (yes)	9 (4%)	6 (9%)	0.106
History	Ascites (yes)	196 (84.5%)	54 (77%)	0.226
	Variceal Bleeding (yes)	91 (39%)	24 (34%)	0.440
	Hepatic Encephalopathy (overt)	43 (18%)	12 (17%)	0.187
	Esophageal varices grade (no/1/2/3/4)	21/79/79/34/5 (12%/35.5%/35.5%/16%/2%)	12/23/19/9/2 (18.5%/35.5%/29%/14%/3%)	0.320
Scores	MELD^c^ before TIPS	12 (6–27)	11 (6–21)	0.036*
	MELD-Na before TIPS	23 (19–32)	22 (19–28)	0.038*
	Child–Pugh score before TIPS	9 (5–14)	9 (5–13)	0.041*
Hepatic hemodynamics	PV-pressure^d^ before TIPS	26 (11–50)	27 (13–46)	0.277
	PSPG^e^ before TIPS	20 (3–38)	19 (11–42)	0.513
	PV-pressure after TIPS	18 (8–34)	20 (8–43)	0.113
	PSPG after TIPS	8 (1–24)	8 (2–36)	0.791
Base laboratory	Sodium (mmol/l)	139 (117–187)	136.5 (115–146)	0.067
	Creatinine (mg/dl)	1.16 (0.45–9.56)	0.99 (0.43–7.61)	0.084
	Bilirubin (mg/dl)	1.005 (0.13–9.51)	1.07 (0.16–6.31)	0.985
	INR^f^	1.2 (0.9–2.5)	1.1 (0.9–2.2)	0.062
	AST^g^ (U/l)	40 (11–1585)	37 (6–83)	0.143
	ALT^h^ (U/l)	25 (8–608)	27 (6–118)	0.816
	Albumin (g/l)	28.2 (3.2–47.6)	30.4 (3.2–49.7)	0.454
	WBC^i^ (10^3^/µl) (Leuko g/l)	6.5 (1.26–50.76)	5.675 (1.3–16.58)	0.01**
	Platelets (× 10^9^/l) (Thrombo g/l)	132 (29–697)	127 (19–509)	0.523

TIPS^a^: Transjugular intrahepatic portosystemic shunt; HCC^b^: hepatocellular carcinoma; MELD^c^: model of end stage liver disease; PV-Pressure^d^: portal venous pressure (mmHg); PSPG^e^: portosystemic pressure gradient (mmHg); INR^f^: international normalized ratio; AST^g^: aspartate aminotransferase; ALT^h^: alanine aminotransferase; WBC^i^: white blood cell count.

*p < 0.05, **p < 0.01, ***p < 0.001.

Overall survival between the groups was not significantly different [n = 154 (65%) dysfunction-free group vs 45 (64%) dysfunction group; p = 0.946, (Table 1)] at a median follow up time of twelve (0–131) months after TIPS implantation. Five dysfunction-free patients (2%) and one patient with TIPS-dysfunction (2%) required liver transplantation. Of the six patients who underwent liver transplantation (LT), the cranial stent end was placed in the hepatic vein in three cases and in the IVC in one case. The other two patients who required LT did not have images of sufficient quality for measurement. TIPS stent did not interfere with LT surgery in any case.

### 2D TIPS geometry measured in digital subtraction angiography

**Figure 3:**
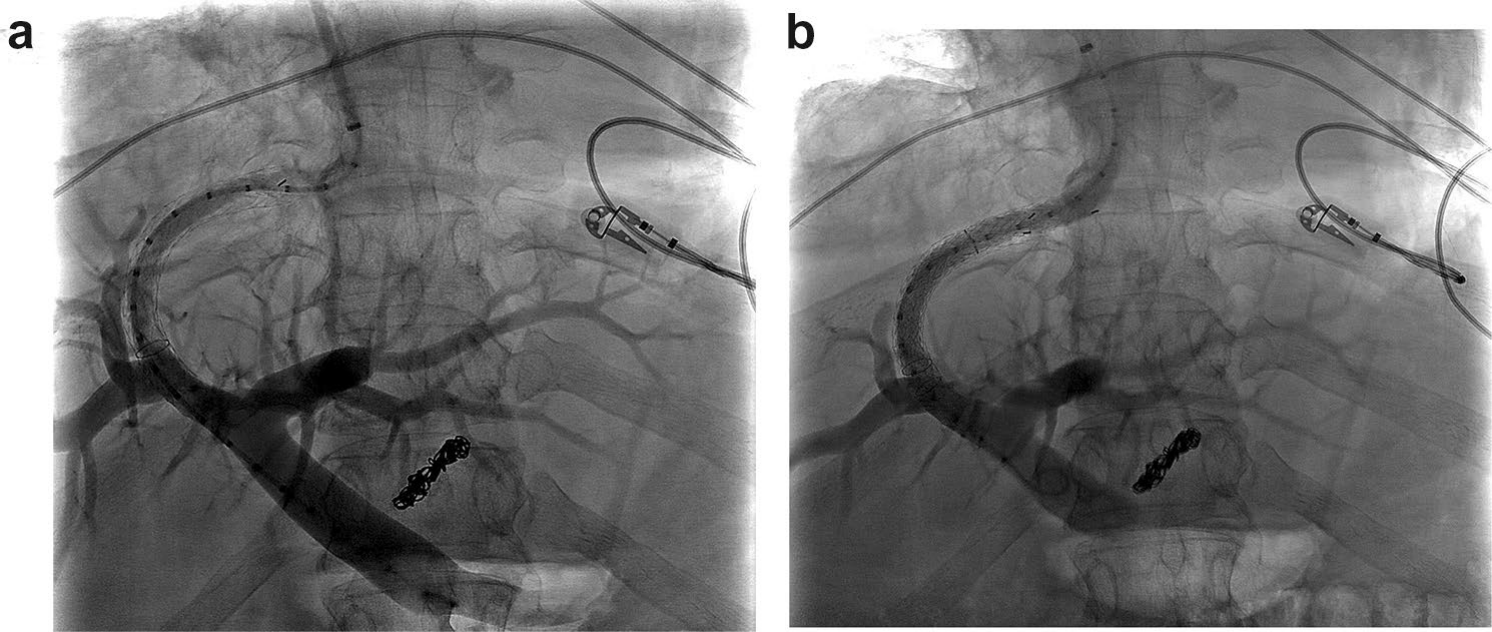
(**a**) Image of 2D DSA projection shows TIPS dysfunction with stenosis at the cranial stent end. DSA performed before TIPS revision. (**b**) Image of 2D DSA projection, performed after successful TIPS revision and extension of the cranial stent end.

In the dysfunction group, the cranial stent end was significantly more often located in the hepatic vein short of the IVC and venous confluence (defined as > 1 cm from the IVC; n = 21 (50%) dysfunction group vs n = 45 (30%) dysfunction-free group, p = 0.014) (Fig. 3a,b). The median distance to IVC in the dysfunction-free group and in the dysfunction group was 0.4 cm (− 4 cm–2.5 cm) and 0.5 cm (− 1.5 cm–3 cm) (p = 0.05), respectively. Interobserver agreement for cranial TIPS position was excellent with an ICC of 0.994 (0.983–0.998) for cranial stent end measurement (Supplementary Table 2). Nominal stent diameter was slightly smaller in dysfunction group (10 mm (10 mm–10 mm) dysfunction-free group vs 10 mm (8 mm–10 mm) dysfunction-group). Other general stent characteristics were not significantly different between the two groups, neither nominal stent length [70 mm (40 mm–80 mm) vs 70 mm (50 mm–80 mm)] nor diameter of balloon dilatation [8 mm (0 mm–10 mm) vs 8 mm (0 mm–10 mm)]. TIPS geometry related parameters such as angles α, β, γ and δ showed no significant difference between the TIPS dysfunction and dysfunction-free group (Table 2); at the same time ICC showed a high interobserver agreement for these parameters (Supplementary Table 2).

**Table 2:** TIPS geometry parameters of angiography.

Parameter		No dysfunction (n = 237)	TIPS dysfunction (n = 70)	p
Stent characteristics	Nominal stent length (mm)	70 (40–80)	70 (50–80)	0.948
	Nominal stent diameter (mm)	10 (10–10)	10(8–10)	0.008**
	Dilatation stent (mm)	8 (0–10)	8 (0–10)	0.383
	Underdilated (yes)	200 (87%)	56 (82%)	0.383
Digital subtraction angiography	Distance cranial TIPS^a^ stent end to IVC^b^ (cm)^c^	0.4 (− 4–2.5)	0.5 (− 1.5–3)	0.05
	Cranial TIPS stent end in hepatic vein^d^	45 (30%)	21 (50%)	0.014*
	Contrast medium reflux into IVC after TIPS implantation	73 (33%)	23 (38%)	0.510
	Retrograde intrahepatic perfusion of the PV^e^ after TIPS implantation	119 (54%)	32 (52.5%)	0.833
	Beginning of covered stent part (PV/Intersection/Liver Parenchyma)	33/179/5 (15%/83%/2%)	6/54/1 (10%/88%/2%)	0.262
	α Angle^f^	29.7 (0.7–279)	31.55 (0.9–279)	0.147
	β Angle^g^	153.65 (0–178.5)	159.7 (0–178.8)	0.275
	γ Angle^h^	152.6 (60.2–179.3)	150.8 (41.4–179.1)	0.363
	δ Angle^i^	128.5 (47.6–174.6)	131.3 (43.2–171.2)	0.959
	Largest SPSS^j^ (no SPSS/umbilical/coronary/splenorenal/other)	1/32/92/30/59 (0.5%/15%/43%/14%/27.5%)	1/5/31/15/15 (1.5%/7.5%/46%/22.5%/22.5%)	0.671
	Any SPSS embolized	104 (50%)	17 (26%)	0.001**

TIPS^a^: Transjugular intrahepatic portosystemic shunt; IVC^b^: inferior vena cava; Distance cranial TIPS stent end to IVC (cm)^c^: negative values indicate that the stent end extends into the IVC; Cranial TIPS stent end in hepatic vein^d^: defined as > 1 cm distance from IVC in DSA; PV^e^: portal vein; α Angle^f^: left TIPS-tract angle beginning at covered stent part to PV in DSA (degrees); β Angle^g^: Angle of cranial TIPS stent end to hepatic vein/ IVC in DSA (degrees); γ Angle^h^: right TIPS-tract angle beginning at covered stent part to PV in DSA (degrees); δ^i^: Distal TIPS-tract angle to PV in DSA (degrees); SPSS^j^: spontaneous portosystemic shunt.

*p < 0.05, **p < 0.01, ***p < 0.001.

Most common landing zone of the covered portion of the TIPS stent were the junction of PV (portal vein) and liver parenchyma (n = 179 (83%) dysfunction-free group; n = 54 (88%) dysfunction group, p = 0.262) as well as the PV lumen (n = 33 (15%) dysfunction-free group; n = 6 (10%) dysfunction group, p = 0.262) (Table 2). Besides, most patients in both groups had SPSS (n = 213 (90%) dysfunction-free group, n = 66 (94.5%) dysfunction-group). Flow measured by Doppler ultrasound 7 days after TIPS procedure showed no significant difference between patients with embolized and persisting SPSS; neither for the portal vein main trunk (43 cm/s (16–81 cm/s) SPSS embolized vs. 44 cm/s (17–87 cm/s) persisting SPSS; p = 0.391), nor for the mean flow in the TIPS tract (94.75 cm/s (3–142 cm/s) SPSS embolized vs. 100 cm/s (3.90–177.33 cm/s) persisting SPSS; p = 0.143).

### Predictors of TIPS dysfunction

In univariable regression analysis, the only TIPS geometry parameters associated with the development of TIPS dysfunction were the position of the cranial stent end in the hepatic vein and the distance of the cranial TIPS stent end from the IVC (Table 3). In multivariable regression models, only the position of the cranial stent end in the hepatic vein showed to be an independent predictor of development of TIPS dysfunction (Table 4). Moreover, the embolization of competing SPSS was also significant in univariable and multivariable regression analysis. None of the measured angles (α, β, γ, δ) or the nominal stent diameter showed a significant association with the development of TIPS dysfunction as well as local processes such as HCC (Table 3). In a subgroup analysis, cases with a stent end < 1 cm from the IVC were divided into those stents ending in the hepatic vein and those ending in the IVC (n = 10 (42%) group with dysfunction vs. n = 48 (45%) group without dysfunction, p = 0.749), showing no significant difference between the two subgroups (univariable regression analysis: p = 0.748, HR 0.863, 95% CI 0.352–2.117)**.**

**Table 3:** Univariable regression analysis with TIPS dysfunction as endpoint.

Parameter	p	OR^a^	95% CI^b^
Cranial TIPS^c^ stent end in hepatic vein^d^	0.015*	2.378	1.183–4.778
Any SPSS^e^ embolized	0.001***	0.360	0.195–0.665
Distance cranial TIPS stent end to IVC^f^ (cm)^g^	0.047*	1.401	1.005–1.952
Nominal stent diameter (mm)	0.999	0.0	0.000 -0.000
Indication for TIPS (variceal bleeding / refractory ascites)	0.535	1.172	0.709–1.938
PV-Pressure^h^ before TIPS	0.321	1.022	0.979–1.068
PSPG^i^ before TIPS	0.492	1.017	0.969–1.068
PV-Pressure after TIPS	0.043*	1.050	1.002–1.101
PSPG after TIPS	0.332	1.031	0.970–1.096
MELD^j^ before TIPS	0.018*	0.922	0.862–0.986
Child–Pugh score before TIPS	0.031*	0.843	0.722–0.985
Contrast medium reflux into IVC after TIPS implantation	0.509	1.219	0.677–2.192
Retrograde intrahepatic perfusion of the PV^k^ after TIPS implantation	0.832	0.941	0.534–1.658
Beginning of covered stent part (Liver parenchyma/PV/TIPS tract)	0.297	0.675	0.322–1.413
α Angle^l^	0.099	1.003	0.999–1.006
β Angle^m^	0.095	1.003	0.999–1.007
γ Angle^n^	0.237	0.994	0.983–1.004
δ Angle^o^	0.734	0.998	0.986–1.010
Sodium (mmol/l) before TIPS	0.142	0.968	0.927–1.011
Creatinine (mg/dl) before TIPS	0.131	0.732	0.488–1.097
Bilirubin (mg/dl) before TIPS	0.761	0.962	0.748–1.236
INR^p^ before TIPS	0.130	0.322	0.074–1.396
ALT^q^ (U/l) (GPT) before TIPS	0.282	0.994	0.983–1.005
Albumin (g/l) before TIPS	0.564	1.007	0.983–1.032
WBC^r^ (10^3^/µl) (Leuko g/l) before TIPS	0.014*	0.894	0.817–9.78
Platelets (× 10^9^/l) (Thrombo g/l) before TIPS	0.674	0.999	0.996–1.003
HCC^s^ before TIPS (yes)	0.411	0.415	0.051–3.375
HCC developed after TIPS (yes)	0.115	2.365	0.811–6.891

OR^a^: odds ratio; 95%-CI^b^: 95% confidence interval; TIPS^c^: Transjugular intrahepatic portosystemic shunt; Cranial TIPS stent end in hepatic vein^d^: defined as > 1 cm distance from IVC in DSA; SPSS^e^: spontaneous portosystemic shunt; IVC^f^: inferior vena cava; Distance cranial TIPS stent end to IVC (cm)^g^: negative values indicate that the stent end extends into the IVC; PV-Pressure^h^: portal venous pressure (mmHg); PSPG^i^: portosystemic pressure gradient (mmHg); MELD^j^ : model of end-stage liver disease; PV^k^: portal vein; α Angle^l^: left TIPS-tract angle beginning at covered stent part to PV in DSA (degrees); β Angle^m^: Angle of cranial TIPS stent end to hepatic vein/ IVC in DSA (degrees); γ Angle^n^: right TIPS-tract angle beginning at covered stent part to PV in DSA (degrees); δ Angle^o^: Distal TIPS-tract angle to PV in DSA (degrees); INR^p^: international normalized ratio; ALT^q^: alanine aminotransferase; WBC^r^: white blood cell count; HCC^s^: hepatocellular carcinoma.

*p < 0.05, **p < 0.01, ***p < 0.001.

**Table 4:** Multivariable regression analysis with TIPS dysfunction as endpoint.

Parameter	p	OR^a^	95% CI^b^
Cranial TIPS^c^ stent end in hepatic vein^d^	0.027*	2.300	1.101–4.806
Any SPSS^e^ embolized	0.006**	0.319	0.140–0.725
MELD^f^ before TIPS	0.119		
PV-Pressure^g^ after TIPS	0.405		
WBC^h^ (10^3^/µl) before TIPS	0.213		

Parameter	p	OR	95% CI
Cranial TIPS stent end in hepatic vein	0.027*	2.300	1.101–4.806
Any SPSS embolized	0.006**	0.319	0.140–0.725
Child-Pugh score before TIPS	0.088		
PV-Pressure after TIPS	0.405		
WBC (10^3^/µl) before TIPS	0.213		

OR^a^: odds ratio; 95%-CI^b^: 95% confidence interval TIPS^c^: Transjugular intrahepatic portosystemic shunt; Cranial TIPS stent end in hepatic vein^d^: defined as > 1 cm distance from IVC in DSA; SPSS^e^: spontaneous portosystemic shunt; MELD^f^: model of end-stage liver disease; PV-Pressure^g^: portal venous pressure (mmHg); WBC^h^: white blood cell count.

*p < 0.05, **p < 0.01, ***p < 0.001.

## Discussion

This monocentric study demonstrates that the position of the cranial stent end in the hepatic vein, measured at the time of TIPS procedure, can predict TIPS dysfunction.

In recent years, several studies evaluated predictors of TIPS dysfunction. Besides obviously identifying the use of bare metal stents as predictors^10,12,16^, other factors such as liver function (MELD)^15^, indication for TIPS procedure^8,12^, interventionalist’s experience^18^ and portal venous flow post TIPS^19^ have been described. Few studies evaluated TIPS geometry, such as stent position^17,20,21,30^, or stent-to-PV angle as predictive parameters of shunt dysfunction. Of all the proposed parameters, our study highlights the importance of the cranial stent position. However, we acknowledge only fair discrimination in our ROC analysis, which may indicate that development of TIPS dysfunction is a multifactorial process and may include the persistence of SPSS as well as other factors. All other suggested parameters of TIPS geometry and angles do not add predictive value, which is in line with smaller series that could not confirm those TIPS geometry parameters as predictors for TIPS dysfunction either^28,31^. Our results did not show increased portal blood flow by embolization of SPSS, which would have been a possible explanation for the influence of embolized SPSS on the lower rate of TIPS dysfunction. Since SPSS were embolized at the discretion of the interventionalist in this study, this decision may have been influenced by preferential flow through the SPSS during completion portography. At this point, it should be mentioned that, in order to make a more general recommendation on embolization of SPSS, further studies are needed.

Other not geometry-related factors such as white blood cell count, PV pressure after TIPS procedure, MELD or Child–Pugh Score were not significant in multivariable regression analysis.

Several reasons for the seemingly contrary results can be attributed to a non-standardized TIPS procedure performed across the world^32^.

First, the clinical practice of anticoagulation during and after TIPS procedure is still debated and no general consensus exist. However, this could play an important factor for TIPS dysfunction by in-stent thrombosis.

Second, the imaging techniques between the studies is inhomogeneous^20^, which might contribute to this result. Importantly, CT or magnetic resonance imaging (MRI) scans, for three-dimensional reconstruction, are usually not indicated immediately after TIPS procedure. Therefore, in clinical routine, angiography from the TIPS procedure often is the only available imaging. For this reason, our study focuses on the two-dimensional angiography data. Nevertheless, 3D geometry might reveal significant angular variations that are not visible in two-dimensional angiography, thus further studies are still needed.

Even though the geometric angles of the stent usually cannot be influenced, the cranial stent end position can be easily influenced by the interventionalist’s choice of stent length. Given that the cranial stent position was the only predictive geometric parameter of TIPS dysfunction in our study, the simple advice for interventionalists is to choose a stent length long enough to cover the entire hepatic vein to the IVC. Practically, our study suggests that the distance between the cranial stent end and the IVC in 2D portogram should be less than 1 cm. In making this recommendation however, it must be kept in mind, that a cranial stent end projecting deep into the IVC may complicate liver transplantation; yet, to date, we have not experienced the impossibility of transplantation due to stent placement.

The suggested passive expansion of nitinol stents may cause changes in the stents geometry over time^33^. With the introduction of controlled expansion stents, there may be further differences in TIPS geometry depending on the type of covered stents^34^. Importantly, the cranial position of the stent should be unaffected by potential changes of stent geometry over time.

Even though this is a comprehensive analysis of the largest cohort on this topic so far, there are some limitations. The main limitation is the retrospective and monocentric character of the study limiting its generalizability. The importance of the stent position in the hepatic vein to IVC junction has been proposed in smaller series^22,23^. However, this study is the largest evaluating 2D angiography data from the time of TIPS placement.

## Conclusion

In conclusion, this study demonstrates that the position of the cranial TIPS stent end measured in two-dimensional angiography imaging at the time of TIPS implantation is an independent predictor of the development of TIPS dysfunction. Our study suggests that the distance between the cranial stent end and the IVC in 2D angiogram should be less than 1 cm.

## Supplementary Information

Supplementary Information 1.

Supplementary Figure S1.
