# Supplementary material for: Cranial stent position is independently associated with the development of TIPS dysfunction
Source: Sci Rep. 2022 Mar 3;12:3559. doi: 10.1038/s41598-022-07595-5 (PMC8894460; doi:10.1038/s41598-022-07595-5)
Supplement: Supplementary file 1 — Supplementary Information 1. [file 41598_2022_7595_MOESM1_ESM.docx]

**Supplementary Fig.1** Kaplan Meier one-year survival comparing dysfunction and dysfunction-free group: X axis: time in months, Y axis: cumulative survival; no significant difference in survival within the first 12 months after TIPS procedure between dysfunction-free and dysfunction group (p=0.605); Numbers at Risk dysfunction-free group vs. dysfunction group in time intervals 0 months (209 vs. 70), 3 months (132 vs 45), 6 months (117 vs 36), 9 months (113 vs 33) and 12 months (106 vs 30)

**Supplementary Table 1**. Procedure and revision details

| **Parameter** | **no dysfunction (n=237)** | **TIPS dysfunction (n=70)** | **P** |
| --- | --- | --- | --- |
| Indication for revision (dysfunction sign in duplex/ gastrointestinal bleeding/ ascites/ other) |  | 22/1/32/11 (33%/1.5%/49%/16.5%) |  |
| Type of revision (dilatation/ shunt extension/ lysis) |  | 32/37/1 (46%/53%/1%) |  |
| Complications during TIPS^a^ procedure | 17 (7%) | 4 (6%) | 0.682 |
| Accidental puncture (ascites/ artery/ gall bladder/ bile ducts/ combined) | 2/5/2/2 (1%/2%/1%/1%) | 1/3/0/0 (1.5%/4.5%/0%/0%) | 0.728 |
| Bleeding after TIPS procedure | 12 (5%) | 1 (1.5%) | 0.188 |

TIPS^a^: Transjugular intrahepatic portosystemic shunt

*p<0.05 **p<0.01 ***p<0.001

**Supplementary Table 2**. Interobserver variability for TIPS geometry parameters of angiography

| **Parameter** | ICC^a^ / Cohen's Kappa |
| --- | --- |
| Distance cranial TIPS^b^ stent end to IVC^c^ (cm)^d^ | 0.994 (0.983-0.998) |
| Cranial TIPS stent end in hepatic vein^e^ | 1 |
| Any SPSS^f^ embolized | 1 |
| α Angle^g^ | 0.979 (0.955-0.990) |
| β Angle^h^ | 0.923 (0.827-0.966) |
| γ Angle^i^ | 0.968 (0.931-0.985) |
| δ Angle^j^ | 0.743 (0.439-0.882) |
| Contrast medium reflux into IVC after TIPS | 0.916 |
| Retrograde intrahepatic perfusion after TIPS | 1 |

ICC**^a^** : Intraclass Correlation Coefficient; TIPS^b:^ Transjugular intrahepatic portosystemic shunt; IVC^c^: inferior vena cava; Distance cranial TIPS stent end to IVC (cm)^d^: negative values indicate that the stent end extends into the IVC; Cranial TIPS stent end in hepatic vein^e^: defined as >1cm distance from IVC in DSA; SPSS^f^: spontaneous portosystemic shunt; α Angle^g^: left TIPS-tract angle beginning at covered stent part to PV in DSA (degrees); β Angle^h^: Angle of cranial TIPS stent end to hepatic vein/ IVC in DSA (degrees); γ Angle^i^: right TIPS-tract angle beginning at covered stent part to PV in DSA (degrees); δ Angle^j^: Distal TIPS-tract angle to PV in DSA (degrees)
