# Supplementary figures and images for: Cranial stent position is independently associated with the development of TIPS dysfunction
Source: Sci Rep. 2022 Mar 3;12:3559. doi: 10.1038/s41598-022-07595-5 (PMC8894460; doi:10.1038/s41598-022-07595-5)

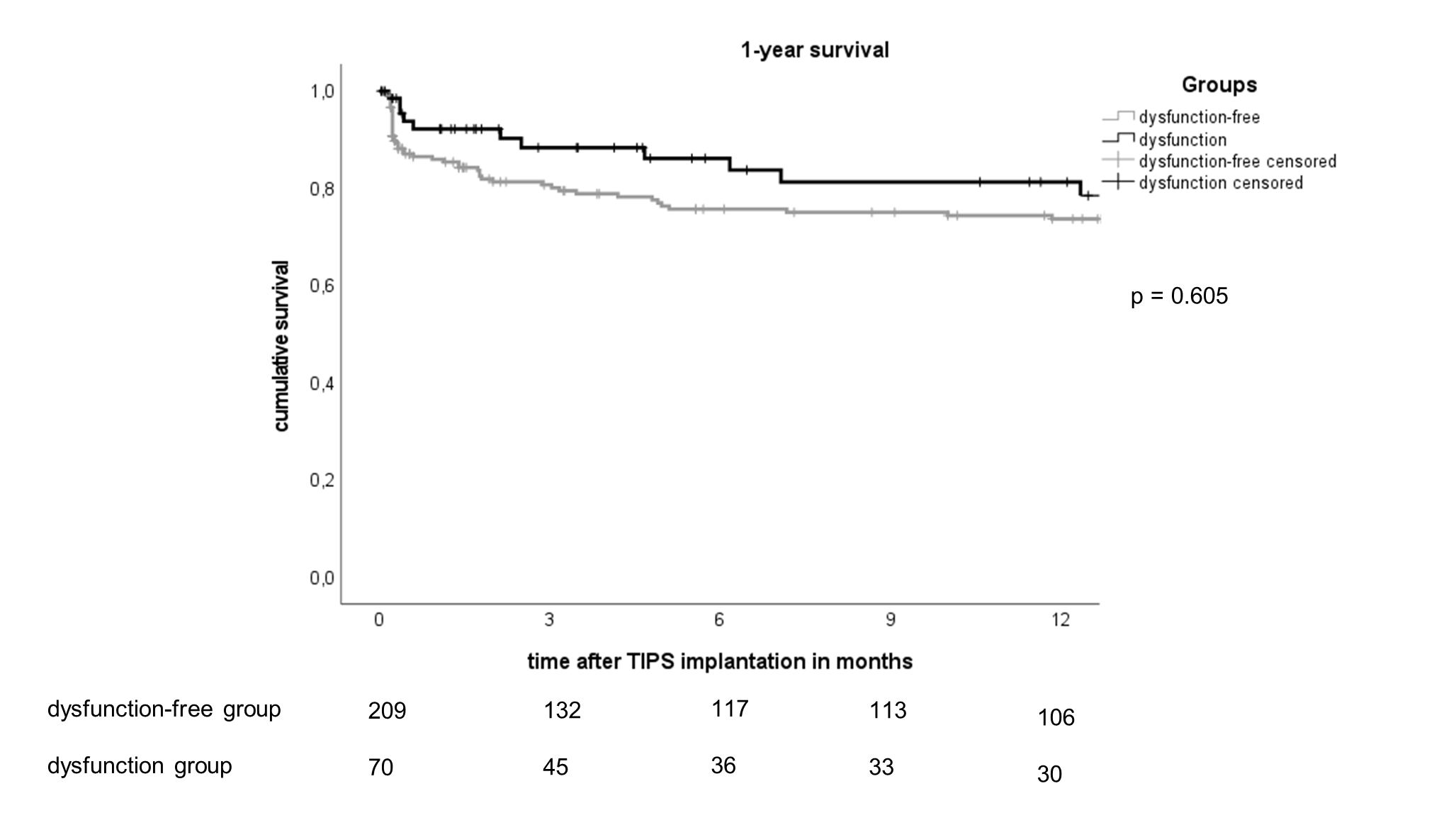

Supplement: Supplementary file 2 — Supplementary Figure S1. [file 41598_2022_7595_MOESM2_ESM.tif]
